# Cholinesterase Inhibitory Activity of Paeoniflorin: Molecular Dynamics Simulation, and In Vitro Mechanistic Investigation

**DOI:** 10.1155/bri/9192496

**Published:** 2024-12-19

**Authors:** Mohnad Abdalla, Asaad Khalid, Jasmine Hedayati, Muhammad Nabeel Ghayur

**Affiliations:** ^1^Pediatric Research Institute, Children's Hospital, Shandong University, Jinan 250022, China; ^2^Substance Abuse & Toxicology Research Center, Jazan University, Jazan 45142, Saudi Arabia; ^3^Medicinal and Aromatic Plants and Traditional Medicine Research Institute, National Center for Research, Khartoum 11111, Sudan; ^4^Kentucky College of Osteopathic Medicine, University of Pikeville, Pikeville 41501, Kentucky, USA

**Keywords:** acetylcholinesterase, Alzheimer's disease, butyrylcholinesterase, docking, enzyme inhibition, memory, Paeoniaceae

## Abstract

Alzheimer's disease (AD), a neurological disorder, is one of the major reasons for memory loss in the world. AD is characterized by a sequela of cognitive and functional decline caused by brain cell degeneration. Paeoniflorin is a monoterpenoid glycoside found in plants of the Paeoniaceae family, which are known for their medicinal properties including dementia. In this project, we report actions of paeoniflorin on the two related cholinesterases (ChE): acetylChE (AChE) and butyrylChE (BuChE). Paeoniflorin, in a dose-dependent (maximum inhibition at 1 mg/mL) manner, inhibited both AChE (0.06–1 mg/mL) and BuChE (0.007–1 mg/mL) enzymes with maximum inhibition of AChE enzyme at 90.3 ± 1.4%, while 99.4 ± 0.3% for BuChE enzyme. The EC_50_ value for the inhibitory effect of the compound against AChE was 0.52 mg/mL (0.18–1.52), while against BuChE was 0.13 mg/mL (0.08–0.21). The observed ani-ChE action was like an effect also mediated by the known ChE blocker physostigmine. Molecular interactions between paeoniflorin and both ChE enzymes were additionally sought via molecular docking and molecular dynamics simulations for 100 ns, that showed paeoniflorin interacted with the active-site gorge of AChE and BuChE via hydrogen bonds and water bridging with the many amino acids of the AChE and BuChE enzymes. This study presents the ChE inhibitory potential of paeoniflorin against both AChE and BuChE enzymes. With this kind of inhibitory activity, the chemical can potentially increase ACh levels and may have use in the treatment of dementia of AD.

## 1. Introduction

Alzheimer's disease (AD), a neurological disease, is one of the major reasons for memory loss in the world [1]. AD is characterized by a sequela of cognitive and functional decline caused by brain cell degeneration. The condition worsens with age and inevitably results in death due to complications of the illness [2]. Currently, there is no confirmed theory for the pathogenesis of AD [3]. However, the disease course is explained by an accumulation of beta-amyloid plaques and hyperphosphorylated tau proteins, inhibiting acetylcholine neurotransmission (ACh) [4]. Most cases of AD present in the early stage of disease progression [5], when patients begin to have problems with concentration, becoming disoriented, and mood changes [6]. The cholinesterase (ChE) family is mainly comprised of acetylChE (AChE) and butyrylChE (BuChE) [7]. ChEs are encoded by separate genes, exhibiting various properties and manifest different physiological importance depending on their species and tissue source [8]. The inhibition of AChE and BuChE were promoted to be an effective approach for managing mild-to-moderate forms of AD [9–11].

Since AD symptoms are caused by lack of ACh [12], symptomatic treatments include donepezil, rivastigmine or galantamine, used for their known role in inhibiting AChE [2]. The appropriate AChE inhibitor is selected by the side effect profile and ease of use [13]. However, it is unclear if there is a benefit in using these agents alone in late-stage illness [14]. In severe cases of AD, the only clinically available pharmacological agent is a chemical known as memantine that works via blocking the excitatory neurotransmitter glutamate [15], which can be used alone or in combination with the AChE inhibitors [16]. Selective serotonin reuptake inhibitors and tricyclic antidepressants can be used to treat the mood and sleep changes experienced by Alzheimer's patients [2].

**Figure 1:**
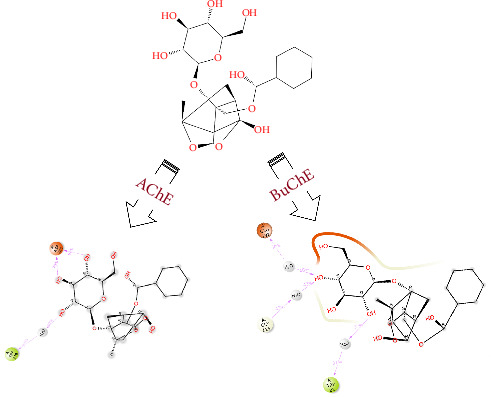
2-Dimensional structure of paeoniflorin (analytical standard; obtained from Sigma-Aldrich, Inc.) and the 2-dimensional interaction pattern observed during 100 ns of MD production run with both enzymes: acetylcholinesterase (AChE) and butyrylcholinesterase (BuChE).

Paeoniflorin is a monoterpenoid glycoside [17]. The chemical structure of paeoniflorin can be seen in Figure 1. Paeoniflorin was first extracted from *Paeonia lactiflora* Pall. (PLP) [17]. PLP has been shown to have anti-inflammatory [18], neuroprotective [19, 20] and beneficial effects in the treatment of AD [21]. Studies have shown paeoniflorin to have activity in depression [17, 22, 23]; epilepsy [24]; neuroprotection [25]; pain and inflammation [26]; atherosclerosis [27]; diabetic wound healing [28], and ability to inhibit inflammatory cytokines in urticaria [29]. Like PLP, paeoniflorin has also been investigated for benefit in cognitive deficits [30] and to down regulate AChE enzyme in vivo in the hippocampus of rats [31].

In this study, we are demonstrating the in vitro ChE inhibitory effect of paeoniflorin against AChE and BuChE, followed by detailed molecular dynamics (MD) simulation, and in vitro mechanistic investigation of enzyme inhibitory effect of paeoniflorin.

## 2. Materials and Methods

### 2.1. Chemicals and Reagents

Following chemicals were obtained, with their supplier: acetylthiocholine iodide (Sigma-Aldrich), butyrylthiocholine chloride (Sigma-Aldrich), 5,5-dithiobis (2-nitro)benzoic acid (DTNB; Sigma-Aldrich), electric eel (type VI-S; Sigma-Aldrich), paeoniflorin (analytical standard; Sigma-Aldrich), physostigmine salicylate (Sigma-Aldrich) and other chemicals used in the enzyme inhibition assay (see below for details). All solutions and dilutions of the said chemicals were made fresh when needed.

### 2.2. AChE and BuChE Inhibition Assays

ChE inhibitory effects were monitored in vitro via a spectrophotometric method that was first reported earlier [32]. The assay process is described already [33, 34], although it is mentioned here in brief. The procedure used SpectraMax microplate spectrophotometer (Molecular Devices, CA, USA) for studying different enzyme inhibitory effects. AChE used in the study is originally from Electric eel (type VI-S) while BuChE is originally from horse serum source. Acetylthiocholine iodide and butyrylthiocholine chloride were employed as substrates in enzyme inhibitory reactions. Ellman reagent, which made up of 5,5-dithiobis (2-nitro) benzoic acid (DTNB), was the main chromogenic marker for measuring ChE effect. Sodium phosphate buffer (1 mM) at pH 7.0 was employed to make enzyme working solution and at pH 8.0 for the assay mixture. All the reactions were in triplicate, and the initial rates were measured as the rate of change in OD/min (optical density/minute) and used in subsequent calculation. Physostigmine was used as a standard ChE inhibitor.

### 2.3. MD Simulation Procedure

Similar studies are reported before in the literature [35–39]. The three-dimensional model of paeoniflorin was constructed using the Dundee PRODRG2 Server [40]. This model was docked in the active-site gorge of ChE using Auto-Dock software [41, 42]. The ChE structure was retrieved from Protein data bank accession I.D. 7B2W, downloaded, protonated and minimized prior to docking. The ligand was considered as a defined ligand-specific torsion tree with flexibility. An Auto-Dock algorithm with precalculated maps of each atom of the ligand and a pre-defined electrostatic potential was utilized. The auto-grid algorithm implemented in Auto-Dock predicted the binding energy, via its ligand conformation and contribution, of each atom of a specified element with the specified grid point in the vicinity of the receptor. Hydrogen atoms were added, and Gasteiger–Marsili charges were calculated using Auto-Dock Tools (https://ccsb.scripps.edu/mgltools, accessed on Sep 10, 2024). Docking results were analysed with Biovia Discovery Studio visualizer 2021, WebLab ViewerPro v.4.0 [43], LIGPLOT v.4.5.3 [44] and LPC software [45].

The postinspected paeoniflorin dock complex in both ChE was further subjected to execute 100 ns MD simulation run using Desmond software. The reason for performing the MD simulations of complexes was for affirming potency of paeoniflorin blockade in AChE and BuChE. Receptor topology was constructed, and SPC water model with specified periodic boundary conditions at a distance of 1.0 nm was standardized to have an aqueous environment. In addition, solvated receptor charges were balanced out with introduction of needed Na^+^ or Cl^−^ ions. Latterly, the default temperature and pressure were retained as suggested in the Parrinello–Rahman algorithm and the Nose–Hoover temperature coupling procedure. Isothermal-isobaric (NPT) ensemble was utilized for minimization and relaxation. MD simulation of 100 runs got accomplished with a time difference of 100 ps. Moreover, trajectories of both complexes were examined/apprehended by plotting the simulation interaction diagram (SID) module present within Desmond Software for purpose of analysing the alterations in terms of stability, variable fluctuations, compactness or the packing of the proteins, electrostatic interactions such as H-bonding interaction with their occupancies. Secondary structural elements (SSE) were also calculated. Ligand properties were also calculated individually by calculating root mean square deviation (RMSD), the radius of gyration (rGyr), torsional angle, molecular surface area (MolSA), solvent accessible surface area (SASA) and polar surface area (PSA). The whole trajectory analysis of 100 ns of each complex was carried out to observe these parameters.

### 2.4. Data Analysis

All results are given as mean ± standard error (“*n*” is the # of observations) and EC_50_ (median effective concentrations) with 95% confidence interval (CI). Stats used: Student's *t*-test and two-way analysis of variance (ANOVA) followed by Bonferroni's multiple comparisons test. These were applied via GraphPad program from San Diego, CA, USA. A *p* value of < 0.05 was taken as significantly different.

## 3. Results and Discussion

### 3.1. AChE and BuChE Inhibition Assays

**Figure 2:**
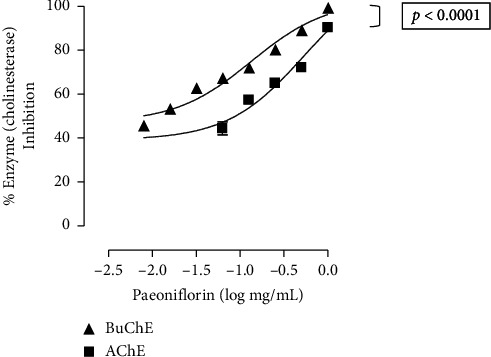
Curves showing % inhibitory effect of paeoniflorin against acetylcholinesterase (AChE) and butyrylcholinesterase (BuChE) enzymes tested via the in vitro enzyme assay for cholinesterase inhibition. Data presented are mean ± SEM, three observations. There is significant different between the two curves, two-way ANOVA followed by Bonferroni's test (*p* < 0.0001).

Paeoniflorin was tested in vitro for its ability to influence AChE and BuChE enzymes. Paeoniflorin, in increasing concentrations, not only inhibited AChE (from 0.06 –1 mg/mL, three observations, Figure 2) but also BuChE (from 0.007–1 mg/mL, three observations, Figure 2). Both the curves were significantly different with the inhibition curve of the chemical against BuChE being more significantly towards the left (*p* < 0.0001, two-way ANOVA). Maximum inhibition exhibited by paeoniflorin against AChE was 90.3 ± 1.4% (three observations, Figure 2), while maximum inhibition on BuChE enzyme was 99.4 ± 0.3% (three observations, Figure 2), both values were significantly different (*p* < 0.0001, Student's *t*-test). The EC_50_ value for the inhibitory effect of the compound over AChE was 0.52 mg/mL (0.18–1.52, three observations), while against BuChE was 0.13 mg/mL (0.08–0.21, three observations). Compared to the effects shown by paeoniflorin, a standard and known mixed-type AChE and BuChE inhibitor physostigmine, showed its enzyme blocking action (data not shown) with EC_50_ values of 0.04 *μ*g/mL (0.04–0.04, *n* of three) and 0.85 *μ*g/mL (0.83–0.10, three observations), respectively.

Paeoniflorin, in the literature, has been reported to have many non-neurological and neurological activities. In studies on PC12 cells after induced oxygen-glucose ischemic-reperfusion injury, paeoniflorin administration increased cell viability and decreased oxidative stress and inflammation by activating the JAK2/STAT3 pathway [46]. After subarachnoid haemorrhage in rats, paeoniflorin also reduced oxidative stress and lessened early brain injury [47], and helped relieve cognitive dysfunction associated with diabetes [48]. Paeoniflorin exhibited neuroprotection for dopaminergic neurons in MPTP mouse representation of Parkinson's disease (PD) [49] and could improve cognitive impairment in PD via inhibition of the JNK/p53 pathway [50]. Most importantly, paeoniflorin is thought to have a therapeutic effect on beta-amyloid plaque deposition [25]. Additionally, as stated above, paeoniflorin was reported some years ago for its cognitive enhancing effects in rats [30] and its ability to reduce AChE levels in vivo in rat brain [31]. In our endeavour, we are demonstrating the in vitro AChE inhibitory effect of paeoniflorin against AChE and over BuChE enzymes. In addition to the known contribution of AChE enzyme in the pathology of AD, BuChE levels are also shown to be augmented in certain brain regions in advanced AD and so targeting BuChE enzyme makes perfect sense pharmacologically [51]. This shows the multidimensional potential of this compound for use in dementia of AD.

### 3.2. MD Simulation Profiling

**Figure 3:**
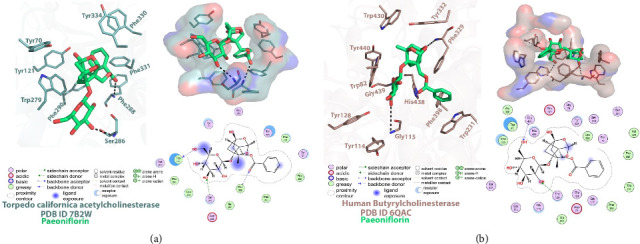
Molecular docking result of paeoniflorin binding into (a) acetylcholinesterase and (b) butyrylcholinesterase enzymes.

**Figure 4:**
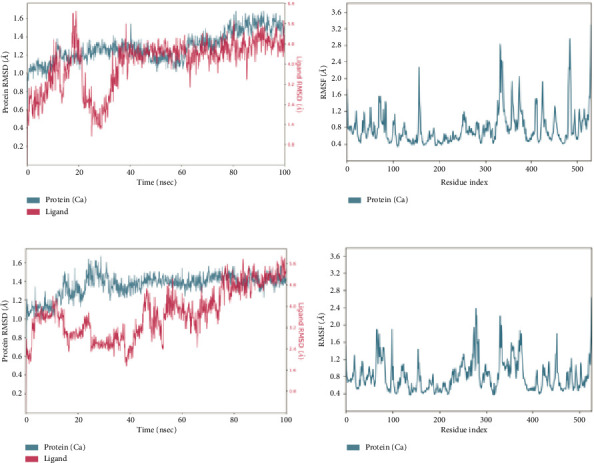
Deviation and fluctuation and lig-fit plots: (a) root mean square deviation (RMSD) and root mean square fluctuation (RMSF) analysis of AChE-paeoniflorin complex, and (b) RMSD and RMSF analysis of BuChE-paeoniflorin complex projected in 100 ns MD run.

**Figure 5:**
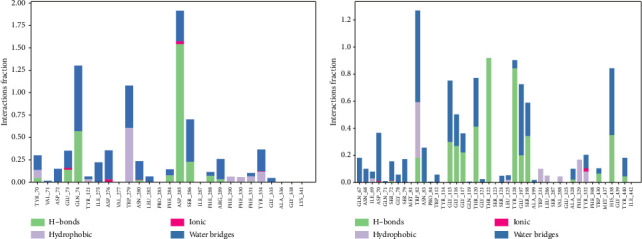
Histogram analysis of the interacted fraction pattern in (a) acetylcholinesterase (AChE) with paeoniflorin and (b) butyrylcholinesterase (BuChE) with paeoniflorin.

**Figure 6:**
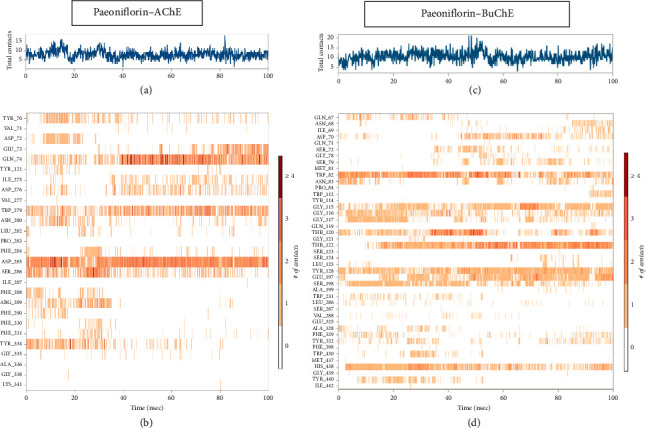
Molecular contact analyses of paeoniflorin against: (a, b) acetylcholinesterase (AChE), and (c, d) butyrylcholinesterase (BuChE), showing: (a, c) A timeline representation of the total no. of molecular interaction contacts in each trajectory frame and (b, d) the number of interactions with the active site residues in each frame of the simulated 100 trajectory frames.

**Figure 7:**
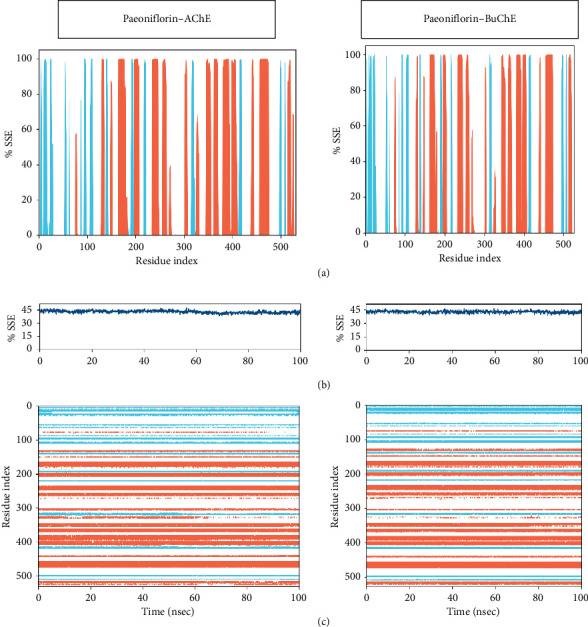
Secondary structural element (SSE) analyses of acetylcholinesterase (AChE) and butyrylcholinesterase (BuChE) complexed with paeoniflorin: (a) % secondary structure elements (SSE) in respect to residue index present in the complex, (b) % SSE in respect to time in nano-scale time frame, and (c) indicates influence of residual change with the variation of projected time frame.

**Figure 8:**
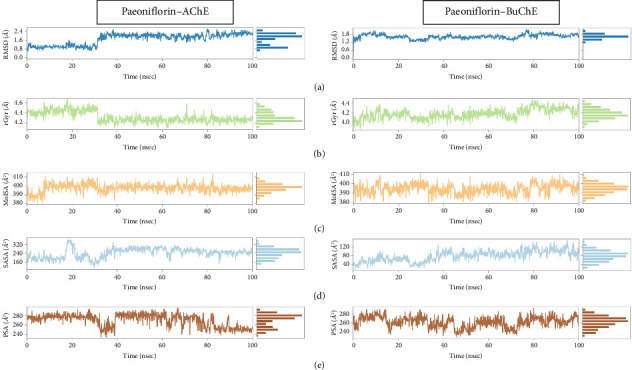
Paeoniflorin property analyses against acetylcholinesterase (AChE) and butyrylcholinesterase (BuChE): (a) ligand root mean square deviation (RMSD), (b) radius of gyration (rGyr), (c) molecular surface area (MolSA), (d) solvent accessible surface area (SASA) and (e) polar surface area (PSA).

Data for molecular docking result of paeoniflorin binding with AChE and BuChE is given in Figure 3 which shows that paeoniflorin interacted with many amino acids of the AChE and BuChE enzymes. The MD simulation results are presented in Figures 4, 5, 6, 7 and 8. The MD simulation interpretation parameters included elemental quantitative analysis to compute paeoniflorin complex constancy in terms of stability, alterations, deviations, and fluctuation of the protein and ligand, in order to check conformational differences during 100 ns of a production run [52]. MD simulation result interpretation is the prime way of numerically assessing to analyse the protein SSE, firmness, stability, inconsistency, variations or differences, elastic stability and molecular recognition analysis between each ChE and paeoniflorin to observe the conformational or translational change during 100 ns of the MD production run.

#### 3.2.1. MD Simulation Analysis of Paeoniflorin in the Vicinity of AChE and BuChE

Stability analyses such as RMSD and root mean square fluctuation (RMSF) are employed in order to determine displacement of atoms present within each ChE during 100 ns MD simulated time. RMSD plot inspected for both receptors of C-*α* backbone atoms and the heavy atoms of paeoniflorin during 100 ns of MD run.

##### 3.2.1.1. RMSD and RMSF Plots With AChE

RMSD plot of AChE-paeoniflorin complex, is shown in Figure 4(a), demonstrates that deviations are present in the spread of 0.9 Å to 1.75 Å during the 100 ns of the trajectories during simulation. Moreover, distortions were observed between 50 ns and 75 ns with the deviation of ±0.7 Å. Ligand fitting RMSD of paeoniflorin within the AChE receptor showed a significant effect in the beginning 10 to 35 ns of the production run lied in the spectrum of 0.4 Å to 6.4 Å. Although, the stability was retained after 25 ns to 100 ns near to ±4 Å. Slight deviation was also observed at 65 ns. Lig fit prot plot demonstrated that ligand has slightly moved further from its binding pocket. RMSF of the protein-ligand complex of 537 amino acid residues was also analysed in 100 ns projected MD simulated time. The Influence of residue fluctuations was also recorded for protein-ligand complex with Tyr70, Tyr121, Trp279, Ser286, Phe288, Phe290, Phe330, Phe331 and Tyr334 (Figure 3(a)). Significant change at Gln74 amino acid residue within the peripheral anionic site (PAS) region was observed. Minor changes were noted with other interacting PAS and an anionic subsite residue.

##### 3.2.1.2. RMSD and RMSF Plots With BuChE

In case of BuChE, paeoniflorin showed receptor deviations in the range of 1.0 Å to 1.6 Å (Figure 4(b)). Deviations were noted between 20 ns and 40 ns with the RMSD difference of ±0.6 Å. Comparing the result of ligand fitting, RMSD with heavy atoms of paeoniflorin in BuChE receptor exhibited big changes during whole MD production run within the spread of 2.0 Å to 5.6 Å. Moreover, the stability was almost achieved after 30 ns to 100 ns near to ±4.8 Å as described in Figure 4(b). Lig fit Prot plot showed that paeoniflorin diffused away from its binding site. The residual fluctuations were also recorded for protein-ligand complex with Trp82, Tyr114, Gly115, Gly116, Thr120, Gly121, Thr122, Tyr128, Glu197, Ser198, Trp231, Phe329, Tyr332, Phe398, Trp430, His438, Gly439 and Tyr440 (Figure 3(b)). The less deviation and fluctuation data suggested that AChE and BuChE when combined with the paeoniflorin, showed stability with the respective protein and its binding region.

#### 3.2.2. Protein-Ligand Contact Analyses During MD Simulation

Stability of the compound paeoniflorin was also measured for both receptor using protein-ligand contact histogram to inspect crucial molecular interactions in the vicinity of active site regions by means of interactions fraction pattern. These protein-ligand molecular interactions were also determined at 100 ns trajectories files of the projected MD production run. These molecular interaction patterns are constituted on hydrogen-bonding and hydrophobic interactions and water-mediating bridges. Hydrogen-bonding interactions play a tremendous representation in computer-aided drug discovery.

##### 3.2.2.1. Protein-Ligand and Molecular Contacts: Paeoniflorin With AChE

Paeoniflorin binding with AChE showed 0.50% and 1.50% prominent hydrogen bonding interaction with Gln74 and Asp285 (Figure 5(a)). Strong hydrophobic interactions were mediated with Trp279 0.55% while less persisted hydrophobic contacts around 0.1% interactions fraction with Tyr70, Tyr121, Phe290, Phe330, Phe331 and Tyr334 were observed. Glu73, Gln74 Trp279 and Asp285 persisted strong H-bond occupancy all over the MD simulation of 100 ns run. Other residues also mediated intra-molecular interactions with insignificant hydrogen bond occupying ability, see shown in Figure 5(a). 2D protein-ligand contact analysis (Figure 1) also indicated that hydroxyl moiety at ortho and meta position of phenol ring present in the paeoniflorin established H-bond with Asp285 persisted to 62% and 75% during 100 ns of the simulated time while another water-bridge interaction mediated with Tyr121 persisted at 30%.

A timeline representation described overall instances of interaction or molecular contacts in each trajectory record frame were also assessed.  Figure 6(a) indicates all the instances of molecular contacts observed within whole simulation. After 40 ns of the simulated trajectory 04 intermolecular contacts persisted in 100 ns scale while the increase or decrease in molecular contacts was recorded at different intervals in MD. The interactions which persisted in each trajectory frame are represented in Figure 6(b). Gln74, Ile275, Asp276, Trp279, Asp285, Ser286 and Phe334, the PAS of gorge residues networked/interacted along the whole time of simulation while Tyr70, Phe75, Gly80, Ser81, Trp84, Leu282, Asp285 and Ser286 did for shorter time and less or invariable molecular interaction observed as shown in pale red colour, which translates as one or dual molecular interaction retained. Moreover, Asp285 represented a dark orange colour band, revealed the highest occupancy with extensive intra-molecular contacts exhibited during the simulation (Figure 6(b)).

##### 3.2.2.2. Protein-Ligand and Molecular Contacts: Paeoniflorin With BuChE

The same inspected analytic parameter utilized in AChE was further applied against BuChE when bound with paeoniflorin (Figures 5 and 6). Protein-ligand contacts indicated that multiple water-bridging reactions mediated with the following residues Gln67, Asn68, Ile69, Asp70, Ser72, Gly78, Ser79, Met81, Asn83, Gly115, Gly116, Gly117, Thr120, Thr122, Tyr128, Gly117, Glu197, Ser198, Ala199, Ala328, Tyr332, Trp430, His438 and Tyr440 with ∼0.1–1.2% of the interacted fractions (Figure 5(b)). Maximum number of hydrogen bonding also observed with various residues of BuChE such as Trp82, Gly115, Gly116, Gly117, Thr120, Thr122, Tyr128, Glu197, Ser198, Trp430, His438 and Tyr440. Strong H-bonding only noted against Thr122 and Tyr128 which were 0.85% and 0.75% of interactions fraction pattern, but afterwards these mediating H-bonding also disappeared and replaced by water-bridging associated interactions. Hydrophobic contributions of Trp82, Trp231, Leu286, Val288 and Tyr332 were also noted but the percentage of interacting fraction was very less (Figure 5(b)). 2D LP-interaction plot also corresponded that Trp82, Gly115 and Glu197 possessed strong intermolecular interaction with paeoniflorin with 37%, 43% and 40% during recorded simulation time represented in Figure 1. The depicted presentation in Figure 6(c) showed that there were 20 intermolecular contacts were reported with the elevation of different type of H-bonding, hydrophobic and Water-mediated bridging interactions. The bottom panel representation of Figure 6(d) explained that Trp82, Thr122, Tyr128, Glu197 and His438 possessed two types of molecular contacts throughout the MD simulation indicated by the band colour. Inconstant number of single molecular contacts was reported with other binding residues of BuChE which have been vanished with respect to time.

#### 3.2.3. SSE Analyses

The protein SSE (P-SSE) were also estimated for each receptor to analyse the secondary structure content of the protein as a function of residue number and of time. The SSE plot fabricated each residue which was shown to contribute to the development of the three basic SSE i.e., strand, helix and loop. AChE showed that helices were found in the abundance and the strands are relatively low as compared to helices indicated in Figure 7(a). The second plot is comprised of two results: Figures 7(a) and 7(b). Top panel represented %SSE with respect to time and there was no dramatic change observed. The result shown persisted in indicating that there were no apparently observable secondary changes after complexation. This result can be observed in the representative data of Figure 7. The residues making helices and strands have the same pace of conformation with no observable changes recorded throughout the simulation. Consequently, the BuChE when complexed with paeoniflorin have inconsiderable changes in the SSE were recorded which is directly reflecting from the observed data illustrated in Figure 7.

The aforementioned result indicated the positional rearrangements were considered via H-bonds occupancy in AChE while water-bridging mediated interactions in BuChE. Moreover, both receptors behaved differently in terms of the molecular contacts. This is shown in the varied and inconsistent number of molecular contacts in the protein-ligand interaction contact analysis with the respective residue of active site and PAS region in both ChE.

#### 3.2.4. Paeoniflorin Stability Profile in Both AChE and BuChE Receptor

The paeoniflorin properties were examined to determine the conformational, transformational, and rotational alterations using pre-MD production data. To exaggerate detailed analysis, RMS deviation, packing or folding mechanism, molecular surface area property using 1.4 Å probe radius, solvent-accessible and Van der Waals surface area calculations of polar elements using PSA, were evaluated for AChE and BuChE receptor after complexation.

##### 3.2.4.1. Paeoniflorin Property Analysis Against AChE

Paeoniflorin showed stability in both receptors but in comparison with AChE, the high-profile stability was observed against BuChE. Paeoniflorin residual fluctuations in the AChE receptor was seen at 35–40 ns encompassing the equilibrium position in the initial 5 ns. The paeoniflorin deviation results were between 0.28 Å to 2.4 Å (Figure 8(a)) while rGyr results suggested varied after 30 ns. The value of rGyr against AChE was in the region of 4.2 Å to 4.6 Å, and the constant result retained around ±4.3 Å (Figure 8(b)). rGyr results demonstrated that after 30 ns of the simulated time paeoniflorin showed more compactness. This persisted until the end of the projected MD trajectory files. Molecular surface area results were also calculated which reflected the stability, although there was a little variation in the initial 30 ns simulation and after 85 ns of the trajectory records it was found unchangeable with the represented area of 400 Å^2^. The range of MolSA was within the approximate value of 380 Å^2^ to 410 Å^2^ as illustrated in Figure 8(c). The SASA result showed inconsistency with the increased value from 160–320 Å^2^ in the initial trajectories of 40 ns simulation. Afterwards the values became constant throughout the whole simulation with the range of 280 Å^2^ (Figure 8(d)). The PSA property showed a deteriorating result. An initial run of MD results suggested that polar O_2_ and N atoms have more significant influence and the highly notable fluctuations which can be seen in Figure 8(e). The PSA value occurred in the range of 240 Å^2^–280 Å^2^ throughout the simulation and considerable fluctuations were noted in thorough simulation.

##### 3.2.4.2. Paeoniflorin Property Analysis Against BuChE

Simultaneously, BuChE receptors showed that paeoniflorin deviation in terms of root mean square was quite reliable than the AChE profile. The results of RMSD projected between 1.2 and 1.8 Å are depicted in Figure 8(a). The values of initial and final trajectories seemed to be in the same plane whereas little distortion was found at the middle section of the projected MD. The measurement of elastic stability or slenderness ratio deviated in the whole projected MD simulation run which fell within the range of 4.0 to 4.4 Å. It clearly demonstrated that over a period of time, the structure became more compacted, as shown in Figure 8(b). Furthermore, surface area measurements focusing on molar, solvent-accessible and polar atoms were also estimated as described in Figures 8(c), 8(d) and 8(e). Molar surface area lied in the spread/range of 380 Å^2^ to 410 Å^2^ was observed as it was in AChE. Fluctuations were noted which were inconstant and varied during the observed time. Subsequently, the SASA result was surprising having great influence of solvent corresponded to strong water-bridge mediating interactions which was previously noted in the protein-ligand histogram contacts as well. The range of SASA was values recorded in the range of 40–120 Å^2^. The polar surface atoms corresponding the value of 240 Å^2^–280 Å^2^ in the MD simulated time and considerable fluctuations were noted in thorough simulation as observed in case of AChE. The studied properties of paeoniflorin demonstrated that changes and noises were observed in the initial or middle part of the projected MD trajectory frames. The MD data revealed that after the initial or middle part of 100 ns of production, run equilibrium position was attained before completion of the simulated time frame. The results on both ChE properties suggested that AChE has a great potency and stability profile than BuChE when complexation with paeoniflorin occur.

#### 3.2.5. Post-MD: Relative Binding Pose Analysis

Molecular docking and post-MD data show the chemical paeoniflorin was able to pierce in the active site gorge region of AChE and BuChE. It is also exhibited invariable molecular interactions in the steady and dynamic state. PAS region aromatic residues of AChE Trp279, Phe290, Phe330, Phe331 and Tyr334 exhibited hydrophobic or pi-pi stacking contacts while Trp279 established interaction with hydroxyl moieties of the phenol group present in paeoniflorin at a distance of 2.83 Å and Asp285 with the distance of 3.0 Å. Post-MD results declared the change in Gln74 emerged when the H-bonding result with 2.03 Å and Asp285 mediated two hydrogen-bonding interactions with the distances of 1.65 Å and 1.87 Å. Asp285, Trp279, Phe331 and Phe334 were determined as persistent interactive residues in both pre- and post-MD stages. Consequently, BuChE showed interaction in the PAS portion of optimized H-bonding with Thr120 achieving a distance of 2.70 Å, Gly115 and Tryr332 while hydrophobic contacts were established with Trp82, Tyr114, Tyr128, Trp231, Phe329, Phe398, Trp430, His438, Gly439 and Tyr440. Thr120 and MD simulation data revealed that Tyr128 possessed hydrogen bonding with the distance of 2.64 Å and 2.73 Å while Thr122 possessed two H-bonds with the distances of 2.24 Å and 2.06 Å. The molecular interaction of Trp82, Phe329 and Tyr332 were also analysed in docked conformation and exhibited hydrophobic contacts. The results displayed that pre- and post-binding residues were the same, but the distances varied with the passage of time and some static interactions converged.

Paeoniflorin has shown significant and worthwhile enzyme inhibitory activity against both AChE and BuChE enzymes. Molecular interactions showed it interacted with the active-site gorge of the enzymes with many amino acids of AChE and BuChE. Most of the currently available medications for AD are AChE inhibitors: donepezil, galantamine and rivastigmine [2]. The duration of use for these AChE inhibitors clinically is limited because there is immense neuronal damage in the disease and AChE levels are at a minimum. Compared to this, the levels of BuChE are highly elevated which is why it plays a key role as a target for reversal of declining ACh levels [53]. Additionally, BuChE has also been shown to be associated with problematic *β*-amyloid deposition in AD, just another reason for a potential benefit of the use of combined AChE and BuChE inhibitors [54]. Multiple studies have shown the importance of BuChE inhibitors for treatment of advanced AD when very little clinical options are available at that level of the disease [55]. One other advantage paeoniflorin offers over some of the available AD meds is its additional potential for disease modification via its multimodal activity, such as influencing other pathways associated with neurodegeneration (e.g., anti-inflammatory and antioxidant properties). It could provide neuroprotection beyond symptomatic relief. This could potentially slow disease progression; something current AChE inhibitors do not accomplish [56]. There is still a long way to go to see this used in the clinical settings. Future endeavours need to focus on pharmacokinetics studies involving brain penetration, duration of action determination, tolerance development, safety profile and potential for interactions before the long process of clinical trials and regulatory issues.

## 4. Conclusion

This study explores the binding mode and the molecular interactions of paeoniflorin with AChE and BuChE complexes. The docking of paeoniflorin with ChEs yielded significant and worthwhile information which is similar to current literature on both AChE and BuChE targets. This ability of the compound to target and inhibit both AChE and BuChE, offers a potentially invaluable option for future therapy in AD. The in-silico approach was used to determine the plausible binding mode of action via the molecular modelling approach, demonstrating the system dynamics of structural traits with the paeoniflorin. MD results suggested that paeoniflorin has noncompetitive binding in both AChE and BuChE. The AChE and BuChE inhibitory properties of paeoniflorin prove its in vitro potential and benefit in the dementia of AD.

## Data Availability

All data from this project are present in this manuscript.
